# Hot Water Treatment Improves Date Drying and Maintains Phytochemicals and Fruit Quality Characteristics of Date Palm (*Phoenix dactylifera*)

**DOI:** 10.3390/foods12122405

**Published:** 2023-06-18

**Authors:** Jianhui Li, Imtiaz Hussain, Muhammad Azam, Muhammad Arslan Khan, Muhammad Tahir Akram, Khalid Naveed, Muhammad Asif, Naveeda Anjum, Jiaoke Zeng, Jiukai Zhang, Hongru Liu

**Affiliations:** 1 College of Chemistry and Materials Engineering, Quzhou University, Quzhou 324000, China; lijianhui@yeah.net; 2 Value Chain Specialist (Dates), Winrock International, Sindh 71000, Pakistan; imtiazhort@gmail.com; 3 Pomology Laboratory, Institute of Horticultural Sciences, University of Agriculture, Faisalabad 38040, Pakistan; marslanuaf@gmail.com (M.A.K.); m.asifroya@uaf.edu.pk (M.A.); 4 Department of Horticulture, PMAS-Arid Agriculture University, Rawalpindi 46300, Pakistan; tahiruaf1966@gmail.com; 5 Department of Plant Pathology, University of Agriculture Faisalabad, Subcampus Depalpur, Okara 56300, Pakistan; khalid.naveed@uaf.edu.pk; 6 Barani Agricultural Research Institute, Chakwal 48800, Pakistan; naveedabari12@gmail.com; 7 Jiangxi Key Laboratory for Postharvest Preservation and Non-Destruction Testing of Fruits & Vegetables, College of Agriculture, Jiangxi Agricultural University, Nanchang 330000, China; jkzeng211@163.com; 8 Agro-Product Safety Research Center, Chinese Academy of Inspection and Quarantine, 11 Ronghua Nanlu, Yi Zhuang, Beijing 100176, China; zhjk_caiq@163.com; 9 Institute of Crop Breeding & Cultivation Research, Shanghai Academy of Agricultural Sciences, Shanghai 201403, China

**Keywords:** tamar stage, Khadrawi, Hillawi, glucose, sucrose, postharvest losses, sensory attributes

## Abstract

Fresh date fruits (cvs. Hillawi and Khadrawi) were harvested at the khalal stage and treated with hot water treatment (HWT) for different time durations (control, HWT-1 min, HWT-3 min, HWT-5 min, and HWT-7 min) to investigate the physicochemical characteristics, phytochemical properties, and sensory attributes. The results revealed that both date cultivars took less time to reach the tamar stage in response to HWT-7 min compared to control. However, Hillawi date fruit showed a higher fruit ripening index (75%) at HWT-3 min, while Khadrawi fruit had a higher ripening index (80%) at HWT-5 min than untreated fruit (10%). Higher weight loss and lower moisture contents were observed in Hillawi (25%) and Khadrawi (20%) date fruit as the immersion period increased in both cultivars. Moreover, soluble solid content was higher in Hillawi (11.77° Brix) in response to HWT-3 min and Khadrawi (10.02° Brix) date fruit immersed in HWT-5 min in contrast with the control group, whereas significantly lower levels of titratable acidity and ascorbic acid content were observed in Hillawi (0.162%, 0.67 mg/100 g) and Khadrawi (0.206%, 0.73 mg/100 g) date fruit in response to HWT (HWT-1 min, HWT-3 min, HWT-5 min, and HWT-7 min) than untreated fruit. Furthermore, noticeably higher levels of reducing sugar (69.83%, 57.01%), total sugar (34.47%, 31.14%), glucose (36.84%, 29.42%), fructose (33.99%, 27.61%), and sucrose (3.16%, 1.33%) were found in hot water-treated Hillawi (immersed for 3-min) and Khadrawi (immersed for 5-min) date fruit, respectively. In addition, total phenolic content, total flavonoids, total antioxidants, and total tannins were substantially superior in date fruits subjected to HWT-3 min (in Hillawi, 128 mg GAE/100 g, 61.78%, 20.18 mg CEQ/100 g) and HWT-5 min (in Khadrawi, 139.43 mg GAE/100 g, 72.84%, and 18.48 mg CEQ/100 g) compared to control. Overall, sensory attributes were recorded to be higher in Hillawi and Khadrawi date fruit after treatment for 3 min and 5 min, respectively. Our findings suggest that HWT is a promising technique that can be adopted commercially to improve fruit ripening and preserved nutritional quality of dates after harvest.

## 1. Introduction

Date palm is a high-value commercial fruit crop that belongs to the family Arecaceae and is commonly cultivated in the hot and dry areas of the world [1]. The total global production of date palm is estimated to be about 9.45 million metric tons [2]. Dates are the third major fruit in Pakistan and among top date producing countries Pakistan ranks in sixth position globally [2]. In some countries, dates are consumed as a staple food due to their high nutritive profile. Date fruits are excellent sources of dietary fiber, vitamins, carbohydrates, and minerals. Whole dates are typically picked and sold at one of three developmental stages: mature firm (bisir or khalal), fully ripe (rutab), and dried (tamar) [3].

Physical and biochemical characteristics of the fruit are the main indicator of fruit quality. However, several changes in physical as well as biochemical attributes upregulate ripening and badly influence the fruit quality. Moreover, owing to the inferior quality and lower commercial value of decayed fruit, a large proportion of dates are commonly wasted; about 1.5 million tons every year are lost during their handling and packaging due to the unavailability of proper postharvest treatments [4]. To reduce losses and improve the quality of date fruit, suitable treatments are required after harvest. Failure to execute these specified postharvest treatments will lead to a significant postharvest loss.

Hot water treatment (HWT) has recently received substantial research attention as a non-chemical and pesticide-free method for simultaneously managing pathogen infestation, alleviating postharvest deterioration, and maintaining fruit quality [5]. HWT plays a crucial role in modulating respiration and ethylene production, which subsequently modify the ripening process, thereby enhancing the organoleptic quality of fruits and vegetables [6]. In addition, HWT treatment induces the host defense response by improving the mechanism of antioxidant enzymes [7] and other antifungal compounds [8]. However, Wall [9] documented that the exposure time of HWT is more effective as compared to the temperature of hot water to delay postharvest senescence and enhance the shelf life of the product. It is reported that HWT substantially maintained quality attributes by stimulating phytochemical and antioxidant potential while altering the ethylene synthesis in banana fruit during the storage period [10].

HWT has been reported to significantly inhibit decay and enhance fruit quality after harvest in a variety of fruits including peach [11], melon [12], strawberry [13], apple, and pear [14,15]. Fallik [16] observed that postharvest HWT effectively reduced fungal spore germination along with improving the overall appearance and quality of fresh vegetables. In addition, HWT notably alleviates chilling injury by reducing lipid peroxidation, improving antioxidant enzymes activities, and regulating cell wall metabolism in mango fruit [17]. Yanclo et al. [18] reported that the application of HWT considerably maintained higher scores for organoleptic characteristics, i.e., color, firmness, taste, texture, and aroma, and prevented the decay incidence by upregulating biochemical changes and modifying respiration in pomegranate. Furthermore, HWT markedly reduced the increase in weight loss and decay incidence and maintained higher firmness, subsequently prolonging the shelf life and improving the quality characteristics of tomatoes [19].

HWT is currently a cost-effective and widely used postharvest protocol to stimulate the ripening process, inhibit decay incidence, and maintain the quality attributes of horticultural produce. Therefore, the aim of the present study was to evaluate the effect of HWT under a specific time duration on the stimulation of ripening and quality attributes of date cultivars Hillawi and Khadrawi.

## 2. Materials and Methods

In our experiment, date fruit (cvs. Hillawi and Khadrawi) were harvested at khalal stage from Experimental Fruit Orchard Square No. 9, Institute of Horticultural Sciences, University of Agriculture, Faisalabad. Disease-free, healthy, and uniformed size fruit were selected and transported to a postharvest laboratory. Dates were sanitized with tween twenty surfactants (0.5%) and sodium hypochlorite (0.3%) for 3 min and after drying at 25 ± 2 °C temperature for 30 min, fruit were randomly divided into five batches. From each batch, 150 fruit (50 fruit per replication) were treated with hot water (65 °C) at different time duration (1 min, 3 min, 5 min, and 7 min). Following hot water treatments, fruits were arranged in plastic boxes and stored at ambient conditions (25 ± 2 °C and 75–80% R.H). Fruits were analyzed for different physical and physiological (time to reach tamar stage (dark brown color, moisture content less the 25%), ripening index, weight loss, and moisture content), biochemical (TSS, TA, ascorbic acid, and sugars), phytochemical (total phenolic contents, total flavonoids and tannin contents and antioxidant activity) and sensory parameters (color, taste, texture, firmness, astringency, and overall acceptability).

### 2.1. Physical and Physiological Parameters

#### 2.1.1. Time Taken to Reach Tamar Stage (DAYS)

To determine the time taken to reach at tamar stage, all treated and untreated date fruits (20 fruit per replication) were visually observed and values were expressed in days.

#### 2.1.2. Ripening Index (%)

The ripening index of date fruit was determined by calculating the ratio between ripe and unripe fruits and the results were described in percentage.

#### 2.1.3. Weight Loss (%)

For weight loss assessment, 20 date fruits (5 fruits per replication) were labeled and weighed using a digital weighing balance (Ek-600, Japan). Fruit weight loss was expressed in terms of percentage with respect to the difference between initial weight and final weight.

#### 2.1.4. Moisture Content (%)

Moisture content of date fruit was observed by using a moisture analyzer (Brabender^®^ MT-C, Duisburg, Germany).

### 2.2. Biochemical Parameters

#### 2.2.1. Total Soluble Solids (Brix%)

Date juice was collected from pitted fruit (5 fruits per replication) by crushing in juice extraction machine (DN-DOB, DEURON, Tokyo, Japan) and was filtered with Whatman paper prior to being employed for analysis. TSS was determined with a hand refractometer (Model DR-A1; ATAGO, Tokyo, Japan), by placing a few drops of filtered juice on the clean prism and results were expressed as degrees of Brix.

#### 2.2.2. Titratable Acidity (%)

To estimate titratable acidity, the 0.1 N NaOH titration method was followed as demonstrated by Wang et al. [20]. For this, 5 mL fruit juice along with 20 mL distilled water was added to a 100 mL beaker. One drop of phenolphthalein indicator was added to the solution and titrated with 0.1 N NaOH until pink color was achieved. TA was expressed in percentage.

#### 2.2.3. Ascorbic Acid (mg 100 mL^−1^)

Ascorbic acid content in date juice was estimated by implementing the Wang et al. [20] standard dichlorophenolindophenol titration approach. To prepare samples, 10 mL date juice and 90 mL oxalic acid (0.4%) were shaken in a 100 mL conical flask. The solution was filtered and 15 mL aliquot from the filtered solution was titrated with dye until a pink shade appeared and persisted at least for a period of 15 s. The amount of ascorbic acid was stated as mg/100 mL of juice.

### 2.3. Estimation of Sugar Contents by HPLC

Sugar components (glucose, fructose, and sucrose) were quantified using the protocol established by Nafees et al. [21] with minor modifications. Briefly, the supernatant was extracted in HPLC grade ethanol (80% *v*/*v*), centrifuged at 13,000× *g*, filtered through 0.45 µm Millipore size membrane filter, and then 10 µL filtered mixture was analyzed in a HPLC (LC-10A HPLC Series, Shimadzu, Kyoto, Japan) equipped with a pump system and refractive index detector (RID-10A). Liquid chromatography separation was performed on a Razex RCM-Monosaccharide Ca^2+^ Phenomenex column using 100% double distilled water as the mobile phase at room temperature (25 ± 2 °C). The flow rate was maintained at 0.60 mL/min with a running time of 30 min. Identified sugars were quantified on the basis of peak areas of external standards consisting of glucose (1%), fructose (1%), and sucrose (1%) solutions. Reducing sugars were calculated as a sum of glucose and fructose and total sugars were calculated by a sum of glucose, fructose, and sucrose. Results were expressed as a percentage of fresh weight.

### 2.4. Phytochemical Assay

#### 2.4.1. Total Phenolic Content (mg GAE/100 g FW)

Total phenolic content assay was performed according to the protocol previously demonstrated by Ainsworth and Gillespie [22] using the Folin–Ciocalteu reagent. The supernatant was extracted, vortexed, incubated, and centrifuged at 15,000× *g*. Lastly, absorbance was recorded at 765 nm and results were reported as mg GAE/100 g fresh weight.

#### 2.4.2. Antioxidant Activity (DPPH Scavenging Activity)

Antioxidant activity was evaluated using 2,2-diphenyl-1-picrylhydrazyl as per the standard described by Aguayo et al. [23] and described as a percentage.

#### 2.4.3. Total Flavonoids

Total flavonoids were measured according to the procedure demonstrated by [24] with slight modifications. The extracted sample (1 mL) was mixed with distilled water (5 mL), sodium nitrite (0.5 mL of, 5%), and aluminum chloride (0.5 mL of 10%). After a 10-min incubation at 25 °C temperature, sodium hydrochloride (5 mL of 1 M) was added to the solution and vortexed. Finally, absorbance of the solution was recorded at 510 nm by using a spectrophotometer (IRMECO, U2020, Lütjensee, Germany) and expressed as mg CE/100 g FW.

#### 2.4.4. Total Tannin Contents

For estimation of tannin contents, a previously established protocol reported by AOAC [25] was employed. Prepared samples of date fruit were titrated with potassium permanganate using indigo carmine as an indicator and results were expressed as a percentage.

### 2.5. Sensory Evaluation

Sensory analysis of date fruit was performed by implementing the protocol suggested by Ismail et al. [26] with some adjustments. Date fruit samples (10 fruits per replication) were arranged in disposable plates and presented to a trained panel of 10 judges (aged 30–40 years). Samples were scored using the hedonic scale ranging from 1 = very poor and 3 = satisfactory to 5 = excellent based on their sensory qualities, i.e., color, taste, texture, firmness, astringency, and overall acceptability.

### 2.6. Statistical Analysis

The present study was carried out according to complete randomized design with two-factor factorial configurations (temperature duration and variety). The data were subjected to the analysis of variance (ANOVA) procedure using Statistix 8.1 (Tallahassee, FL, USA) statistical software, and significant differences among treatment means were calculated using the least significance difference test (*p* ≤ 0.05).

## 3. Results

### 3.1. Time Taken to Reach the Tamar Stage (Days)

**Figure 1:**
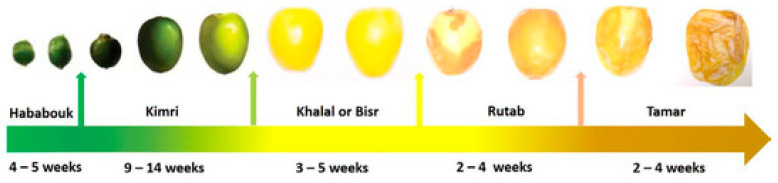
Different developmental and ripening stages of date fruits.

**Figure 2:**
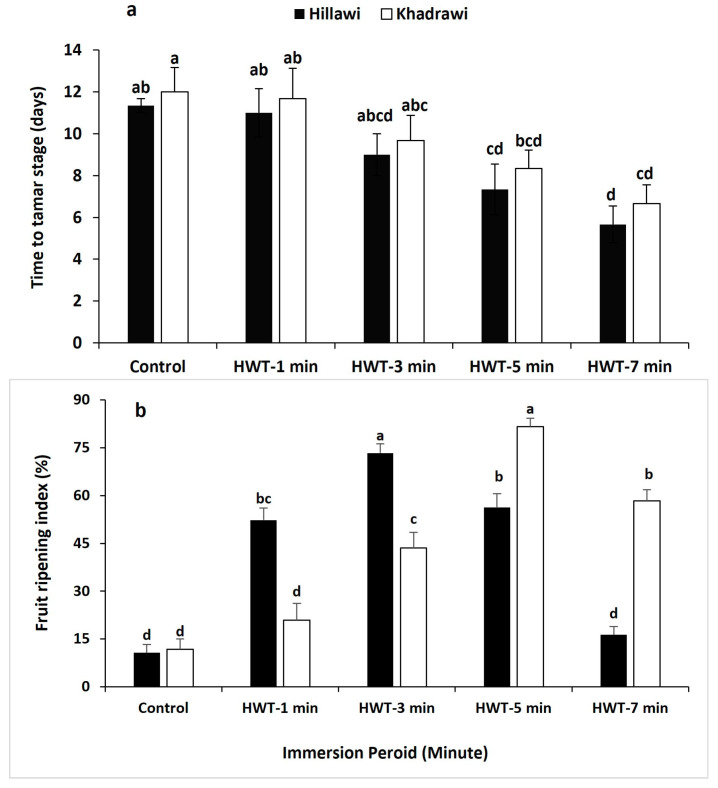
Effect of HWT on time to tamar stage (**a**) and fruit ripening index (**b**) in date palm cvs. Hillawi and Khadrawi. Data collected form mean of four replicates and vertical bars indicate standard error of means. Mean values with different letters show significant differences and same letters indicate no statistically significant difference for all treatments according to the LSD test (*p* < 0.05).

The data pertaining to time taken for the fruit to reach the tamar stage showed that HWT significantly (*p* ≤ 0.05) reduced the time to reach the tamar stage in both date cultivars (Figure 1). However, a control group of both date cultivars took a longer time to reach tamar stage. Moreover, fruit of both date cultivars dipped in hot water (65 °C) for a period of 7 min reach the tamar stage 5 days earlier than the untreated group, whereas the date fruit immersed in hot water (65 °C) for 5- and 3-min duration significantly reduced the period from khalal stage to tamar stage and reached tamar stage 4 and 2 days earlier, respectively, as compared to control fruit (Figure 2a).

### 3.2. Ripening Index (RI)

Ripening index (RI) established a noticeably increasing trend in all treatments of date palm fruits (Figure 2b). However, the ripening index was significantly lower in both untreated control cultivars of date palm fruits. In other durations of hot water treatments, date palm fruit cultivar Hillawi exposed to treatment of 3 min duration possessed a higher ripening index (73.33%) than control fruit. Moreover, HWT-treated date fruits immersed for 5 min showed a maximum ripening index 66.67% higher than that of control fruit.

### 3.3. Weight Loss (WL)

**Figure 3:**
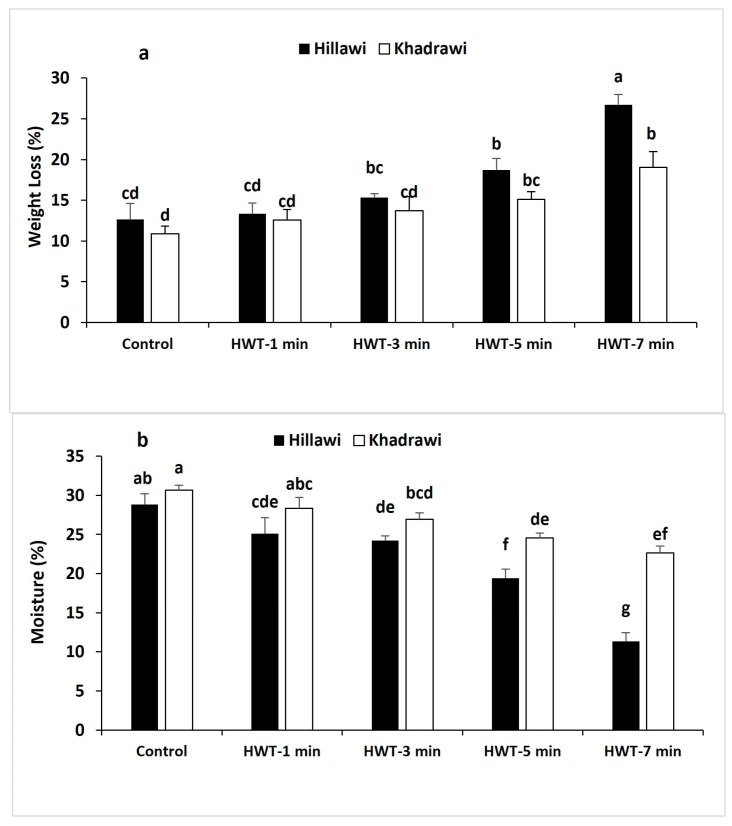
Effect of HWT on weight loss (**a**) and moisture content (**b**) on date palm cvs. Hillawi and Khadrawi. Data collected form mean of four replicates and vertical bars indicate standard error of means. Mean values with different letters show significant differences and same letters indicate no statistically significant difference according to the LSD test (*p* < 0.05).

Physiological weight loss (WL) of date palm fruits significantly progressed in all hot water treatments with the advancement in duration at 65 °C (Figure 3a). Postharvest immersion of hot water for 3 min statistically reduced the PWL of date palm fruits from cultivar Hillawi more (15.34%) than untreated control fruits (*p* ≤ 0.05). However, hot water treatment significantly reduced the increase in WL in the Khadrawi cultivar (15.13%) when subjected to 65 °C for 5 min (Figure 3a). Moreover, lower WL was observed in Hillawi and Khadrawi cultivars exposed to 65 °C for 3 and 5 min, respectively.

### 3.4. Moisture Content (%)

Moisture contents were gradually increased in untreated control Hillawi (28.81%) and Khadrawi fruits (30.66%) exposed to high temperature (65 °C). Mostly, date palm cultivar Hillawi fruit treated with hot water treatment for 3 min at 65 °C had considerably lower moisture content (24.20%) (Figure 3b). However, date palm cultivar Khadrawi immersed in hot water treatment for 5 min exhibited lower TA (24.54%). Moreover, moisture contents were significantly different between Hillawi and Khadrawi fruits treated with hot water for 3 and 5 min (*p* ≤ 0.05).

### 3.5. Soluble Solids Content (SSC)

**Figure 4:**
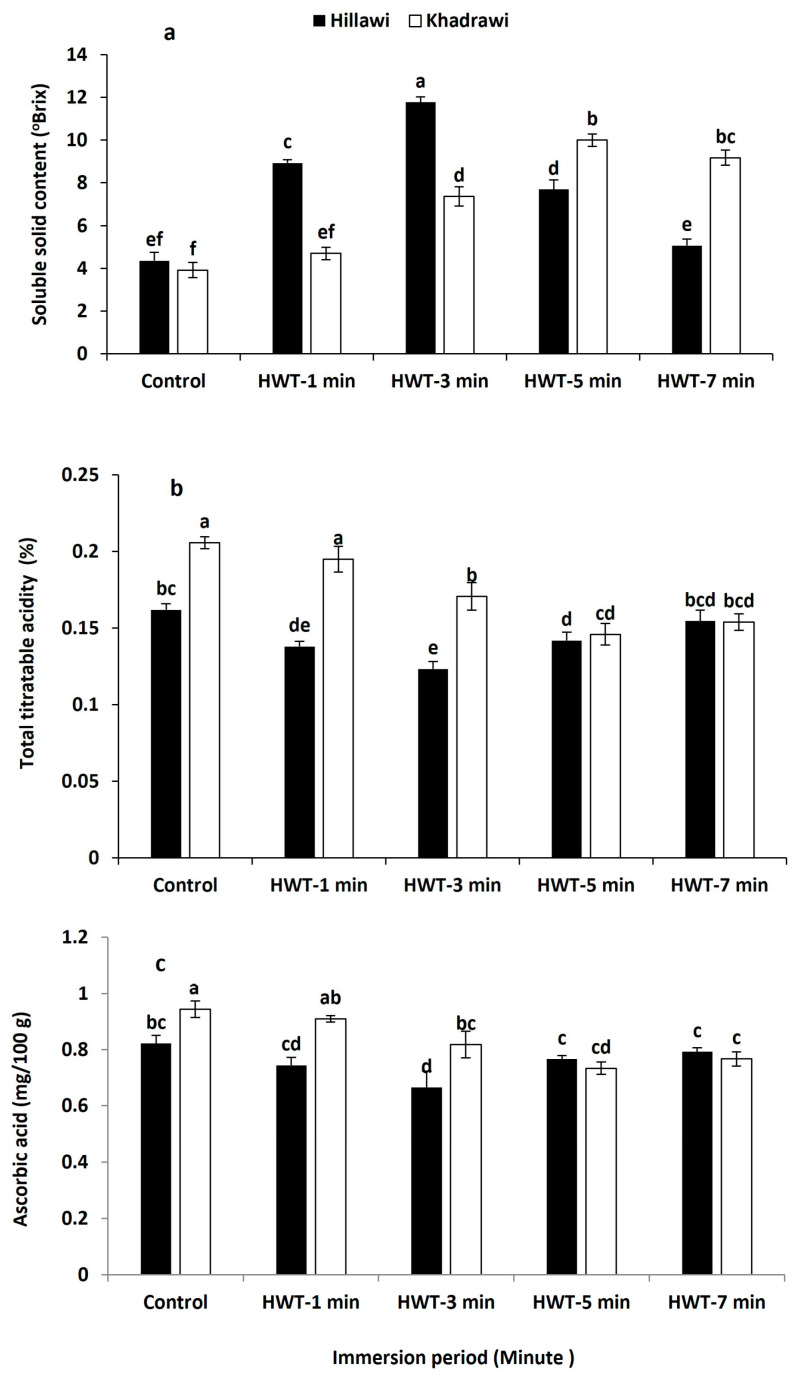
Effect of HWT on soluble solid content (**a**), total titratable acidity (**b**), and ascorbic acid (**c**) on date palm cvs. Hillawi and Khadrawi. Data collected form mean of four replicates and vertical bars indicate standard error of means. Mean values with different letters show significant differences and same letters indicate no statistically significant difference according to the LSD test (*p* < 0.05).

The soluble solids content progressively increased in all hot water treatments when exposed at 65 °C (Figure 4a). Notably higher TSS content was exhibited in date palm fruits of cultivar Hillawi (11.77° Brix) when immersed in hot water for 3 min at 65 °C. Moreover, hot water treatments substantially increased the SSC contents in the Khadrawi cultivar (10.02° Brix) that was treated for 5 min at 65 °C. However, both date palm fruit cultivars, i.e., Hillawi and Khadrawi, treated with hot water for 3 and 5 min at 65 °C showed maximum SSC contents in contrast to untreated date palm fruits (Figure 4a).

#### Titratable Acidity (TA)

TA contents were gradually maintained in untreated control Hillawi (0.162%) and Khadrawi fruits (0.206%) exposed to high temperature (65 °C) (Figure 4b). In general, date palm cultivar Hillawi fruit treated with hot water for 3 min at 65 °C maintained significantly higher TA contents (0.123%). However, fruits of date palm cultivar Khadrawi immersed in hot water for 5 min exhibited maximum TA (0.146%). Moreover, TA contents showed significant difference between Hillawi and Khadrawi fruits treated with hot water for 3 and 5 min (*p* ≤ 0.05).

### 3.6. Ascorbic Acid Content (AA)

Ascorbic acid was significantly reduced in all hot water-treated date palm cultivar fruits subjected to 65 °C (*p* ≤ 0.05) compared to untreated fruits. Generally, untreated control fruits of cultivars of both date palms, i.e., Hillawi and Khadrawi, exhibited maximum contents of AA (Figure 4c). However, date palm cultivar Hillawi possessed 0.67% more AA content when exposed to hot water treatment for 3 min than untreated Hillawi fruits (0.82%). Moreover, fruits of the Khadrawi cultivar of date palm had 0.73% lower AA contents than untreated control fruits (0.94%) after immersion for 5 min. Moreover, ascorbic acid contents of Hillawi and Khadrawi cultivars of date palm showed significant difference to those of untreated control fruits of both cultivars (Figure 4c).

### 3.7. Reducing Sugars (%)

The reducing sugars progressively increased in all hot water treatments in fruits exposed at 65 °C (Table 1). Notably higher reducing sugar contents were exhibited in date palm fruits of cultivar Hillawi (69.83%) when immersed in hot water for 3 min at 65 °C. Moreover, hot water treatments substantially increase the reducing sugar contents in the Khadrawi cultivar (57.01%) fruits subjected for 5 min at 65 °C. However, both date palm fruit cultivars, i.e., Hillawi and Khadrawi, treated with hot water for 3 and 5 min at 65 °C showed maximum reducing sugar contents in contrast to untreated date palm fruits (Table 1).

### 3.8. Total Sugars (%)

Total sugar contents were recorded as significantly (*p* ≤ 0.05) lower in untreated control Hillawi (34.47%) and Khadrawi fruits (31.14%) than those that had HWT treatments (65 °C). Date palm cultivar Hillawi fruit treated with hot water for 3 min at 65 °C had the highest total sugar content (73.04%) (Table 1). However, fruit of the date palm cultivar Khadrawi immersed in hot water treatment for 5 min exhibited maximum total sugar content (59.71%). Moreover, total sugar contents showed significant difference between Hillawi and Khadrawi fruits treated with hot water treatment for 3 and 5 min (*p* ≤ 0.05).

### 3.9. Glucose, Fructose, and Sucrose

**Figure 5:**
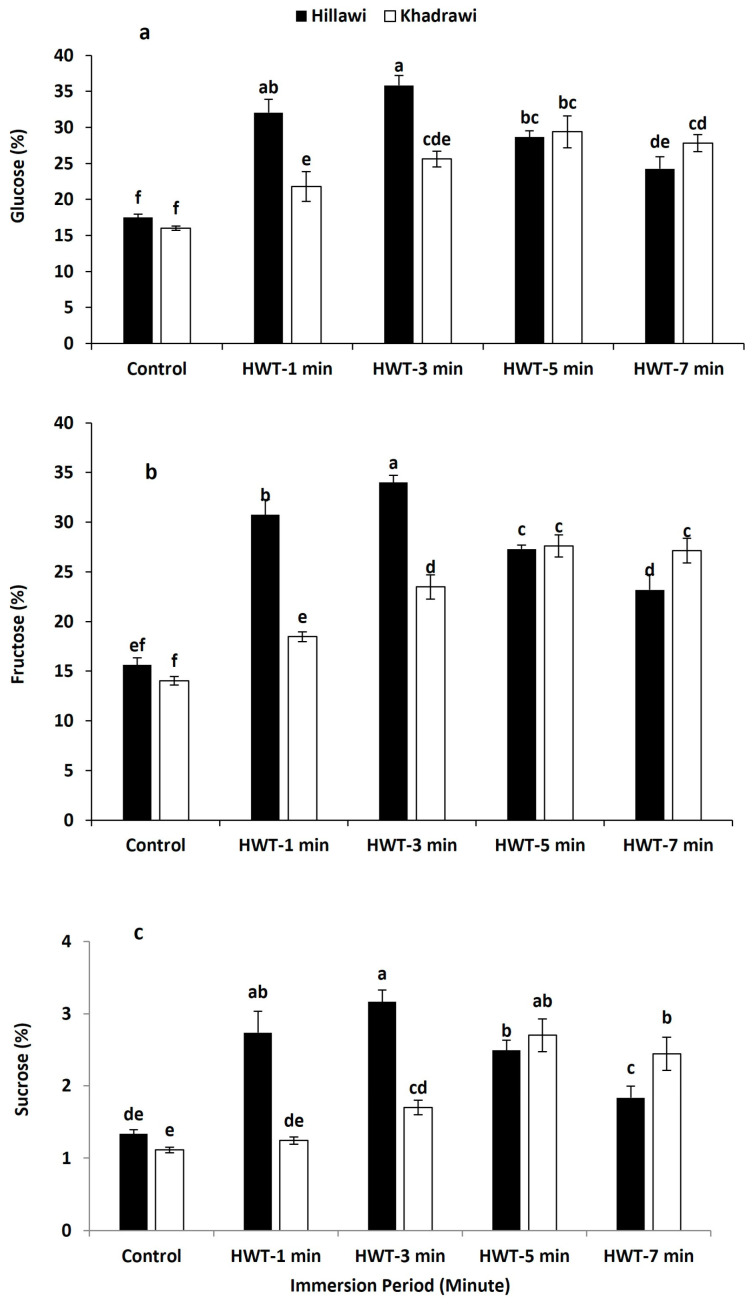
Effect of HWT on glucose (**a**), fructose (**b**), and sucrose (**c**) on date palm cvs. Hillawi and Khadrawi. Data collected form mean of four replicates and vertical bars indicate standard error of means. Mean values with different letters show significant differences and same letters indicate no statistically significant difference according to the LSD test (*p* < 0.05).

The glucose, fructose, and sucrose levels showed a significantly greater increasing trend in all hot water-treated fruits than untreated control fruits of both cultivars of date palm (Figure 5a). However, Hillawi date palm cultivar fruits exhibited maximum glucose level (36.84%) when soaked in hot water for 3 min. Moreover, Khadrawi date palm cultivar fruits possessed a maximum level of glucose (29.42%) when subjected to hot water for 5 min compared to control untreated fruits of the Khadrawi cultivar (17.51%) (Figure 5a). So, the Hillawi date palm cultivar showed a significantly higher maximum glucose level than the Khadrawi date palm cultivar.

Therefore, fructose levels increased in all hot water-treated fruits of both cultivars in contrast to that in untreated control fruits. However, Hillawi fruits treated with hot water for 3 min exhibited a significantly higher level of fructose (33.99%) than untreated control fruits (15.64%) (Figure 5b). Moreover, the Khadrawi cultivar of date palm had a maximum level of fructose (27.61%) when subjected to hot water treatment for 5 min. Consequently, Hillawi date fruit had a higher maximum level of fructose than Khadrawi cultivar date palm fruit (Figure 5b).

Sucrose levels progressed in all hot water-treated fruits of both cultivars of date palm, although Hillawi date palm cultivar fruits exhibited a higher level of sucrose (3.16%) than untreated control fruits (1.33%) when immersed in hot water for 3 min (Figure 5c). Moreover, the maximum sucrose level (2.7%) was observed in Khadrawi cultivar date palm fruits when subjected to hot water for 5 min (Figure 5c). Therefore, the Hillawi cultivar had a significantly higher maximum level of sucrose than Khadrawi cultivar date palm fruits.

### 3.10. Total Phenolic Contents

**Figure 6:**
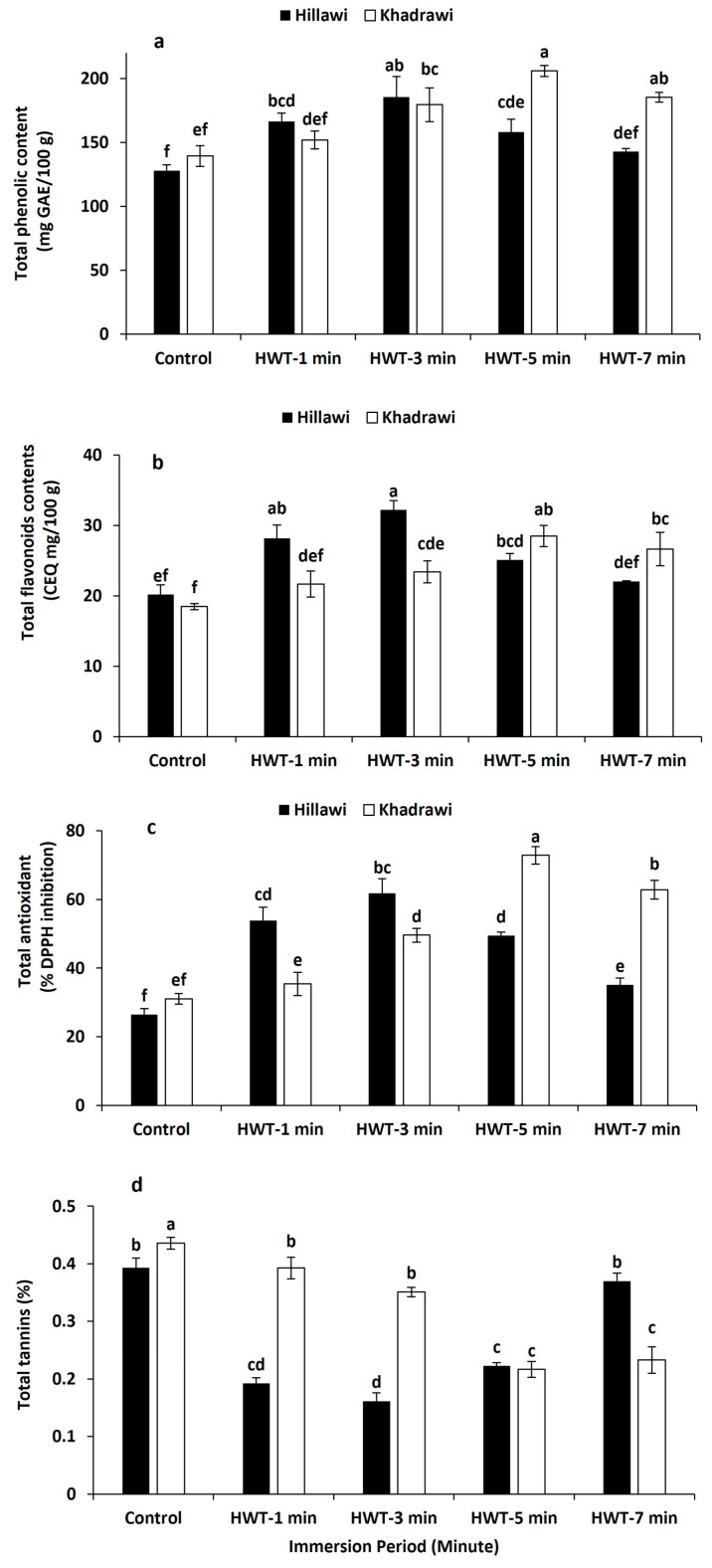
Effect of HWT on total phenolic content (**a**), total flavonoid content (**b**), total antioxidants (**c**), and total tannin content (**d**) on date palm cvs. Hillawi and Khadrawi. The data were recorded from the mean of four biological replications and vertical bars and different letters depict the standard error and significant variation among means. Data collected form mean of four replicates and vertical bars indicate standard error of means. Mean values with different letters show significant differences and same letters indicate no statistically significant difference according to the LSD test (*p* < 0.05).

The TPC content of both cultivars of date palm was significantly higher after all hot water treatments than that of untreated control fruits of both cultivars of date palm (Figure 6a). Overall, TPC contents were considerably lowered in untreated control fruits of Hillawi and Khadrawi (128 mg GAE/100 g and 139.43 mg GAE/100 g). However, Hillawi cultivar date palm fruits had maximum TPC content (185.44 mg GAE/100 g) when soaked in hot water treatment for 3 min. Moreover, fruits of date palm cultivar Khadrawi possessed the highest TPC contents (206.07 mg GAE/100 g) when subjected for 5 min to hot water (Figure 6a).

### 3.11. Antioxidant Activity

Antioxidant activity was notably enhanced in fruits subjected to hot water treatments compared to untreated control fruits of both cultivars of date palm. Higher DPPH activity was observed in fruits immersed in hot water for 3 and 5 min than untreated control fruits (Figure 6b). However, untreated Hillawi and Khadrawi fruits showed greater statistical difference than hot water-treated Hillawi and Khadrawi fruits at 65 °C. On average, Hillawi cultivar date palm fruits exhibited maximum DPPH activity (61.78%) when dipped in hot water for 3 min (Figure 6b). However, Khadrawi fruits had the highest content of antioxidants (72.84%) when immersed in hot water for 5 min.

### 3.12. Total Flavonoids

Total flavonoids and tannin contents of both cultivars of date palm were significantly more progressed in all hot water-treated fruits than untreated control fruits of both cultivars, i.e., Hillawi and Khadrawi (Figure 6c). Overall, flavonoid contents were considerably lowered in untreated control fruits of Hillawi and Khadrawi (20.18 mg CEQ/100 g and 18.48 mg CEQ/100 g). However, Hillawi cultivar date palm fruits showed significantly higher maximum flavonoid contents (32.21 mg CEQ/100 g) when treated with hot water for 3 min. Moreover, Khadrawi cultivar date palm fruits had the highest flavonoid contents (28.53 mg CEQ/100 g) when immersed in hot water for 5 min (Figure 6c).

### 3.13. Total Tannin

Tannin contents of both Hillawi and Khadrawi cultivars of date palm possessed a significantly increasing trend at 65 °C. However, Hillawi date palm cultivar fruits treated with hot water for 5 min exhibited lower tannin contents (0.16%) than untreated control Hillawi fruits (0.39%) at 65 °C (Figure 6d). Moreover, date palm cultivar Khadrawi fruits immersed in hot water for 5 min had 0.22% higher tannin contents than untreated control fruits of Khadrawi (0.39%). So, date palm cultivar Khadrawi possessed higher maximum tannin content than Hillawi cultivar date palm fruits subjected to hot water treatment.

### 3.14. Sensory Evaluation

**Figure 7:**
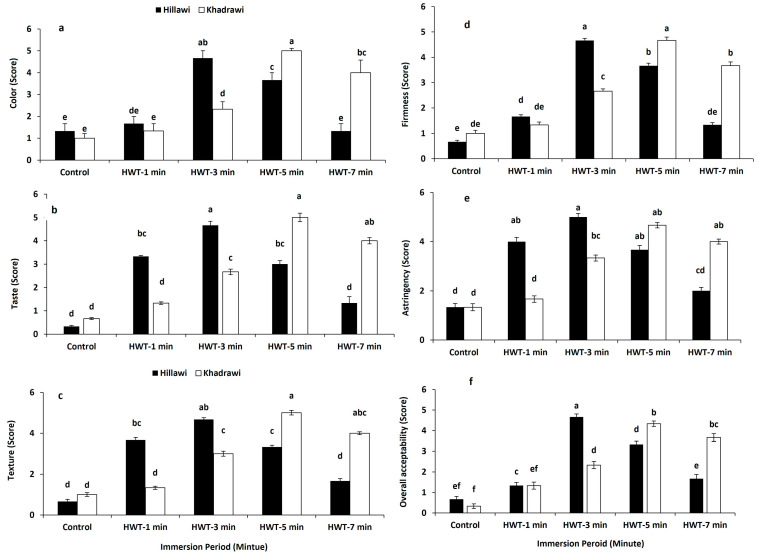
Effect of HWT on color (**a**), taste (**b**), texture (**c**), firmness (**d**), astringency (**e**), and overall acceptability (**f**) on date palm cvs. Hillawi and Khadrawi. Data collected form mean of four replicates and vertical bars indicate standard error of means. Mean values with different letters show significant differences and same letters indicate no statistically significant difference according to the LSD test (*p* < 0.05).

Quality attributes such as color, taste, texture, firmness, astringency, and overall acceptance of both cultivars of date palm fruits were considerably improved after hot water treatment compared to untreated control fruits of both cultivars (Figure 7a–f). However, Hillawi date palm cultivar fruits had a maximum score for color, taste, texture, firmness, astringency, and overall acceptance with values of 4.67%, 4.79%, 4.51%, and 4.67% when subjected to hot water treatment for 3 min. Moreover, fruits of date palm cultivar Khadrawi showed higher values for taste, texture, astringency, and overall acceptance (0.45%, 0.47%, 0.49%, and 0.43%) than untreated control fruits of Khadrawi (0.67%) when immersed in hot water for 5 min (Figure 7a–f).

## 4. Discussion

Fruit ripening is a natural process which involves a number of physiological and chemical changes, making it become softer and palatable. The ripening index is regarded as an essential determinant of fruit flavor as well as consumer acceptability and satisfaction. Accumulation of sugars, decline of organic acid, ethylene synthesis, increased respiration and activation of softening enzymes are the major factors upregulating the ripening process [27]. Overall, the Hillawi and Khadrawi date cultivars treated with hot water for 3 and 5 min at 65 °C showed a higher ripening, respectively, compared to the control group. The higher ripening in hot water-treated dates might be ascribed to higher metabolic activities, lower moisture content, and loss of firmness. In this context, Shahnawaz et al. [28] reported that mango fruit treated with hot water exhibits higher moisture loss and ripened earlier and had higher organoleptic properties than those fruit without heat treatment. Similarly, Jacobi et al. [29] observed that hot water treatment accelerates ripening and regulates color development in mango fruit.

Moisture content plays a crucial role in reducing the deterioration of fresh produce. Generally, a commodity with higher moisture content is extremely susceptible to decay and fruit rot. The results from the present study showed that hot water-treated dates exhibited lower moisture content that might be attributed to the accelerated respiration and transpiration rates. Our results are in agreement with those of a previous study that reported that banana fruit dipped in hot water at 60 °C exhibited lower moisture content due to this regulating the respiration and transpiration rates [30]. Moreover, it was observed that hot water at 70 °C significantly increased the loss of moisture contents in date fruit [31]. Similarly, Ali et al. [32] discovered that hot water-treated date fruits had less moisture content than untreated fruits.

In the current study, hot water treatment effectively maintained higher SSC contents whereas it produced significantly lower TA and ascorbic acid contents. Our results are in agreement with those of Lara et al. [33], who documented that strawberry fruit dipped in hot water at 45 °C for 15 min showed markedly higher SSC content and minimum TA content. Similarly, date fruit hot water-rinsed at 70 °C retained significantly higher SSC content than untreated fruit [31]. Heat treatment induces alteration in monosaccharide and disaccharide metabolism by utilizing organic acid as a substrate during the process of respiration [34]. The higher SSC and lower acidity in hot water-treated date cultivars might be due to modification of sugar and acid metabolism. Our results are also in accordance with previous findings suggesting that higher SSC and lower TA contents were found in hot water-treated banana [30], strawberry [35], tomato, and apple [36]. Ascorbic acid has comparatively lower stability and is more likely to decrease in fruits and vegetables subjected to heat treatment [37]. Consistent with our results, heat treatment significantly reduced the level of ascorbic acid in selected vegetables [38] spinach leaves [39] and bitter melon [37]. The loss of ascorbic acid in hot water-treated date cultivars might be associated with higher moisture loss and accelerated metabolic activities.

Sugar accumulation in fruit determines the taste and sweetness and is one of the most crucial parameters in fruit quality [40]. Glucose, fructose, and sucrose are the major sugars found in date flesh [41]. In sugar metabolism, the breakdown of sucrose is followed by increases in glucose and fructose during the ripening phase [42]. Abidi et al. [43] documented that higher sucrose and glucose levels are directly associated with a higher sensory quality of peach during storage. Likewise, Wang et al. [44] suggested that a higher level of sucrose plays a key role in improving membrane firmness and overall acceptability of peach fruit. In the current experiment, HWT produced higher sugar content in both date cultivars. In the same context, Holland et al. [45] documented that heat treatment showed significantly higher accumulation of sugar content and improved the palatability of citrus fruit. Our results are also consistent with earlier studies that found HWT produced notably higher sugar contents and produced better flavor in peach [46] and banana [30]. The mechanism behind the increased sugar content after applying HWT could be ascribed to activation of glucosidase, galactosidase, and arabinose enzymes due to higher catabolism. Similarly, a higher sugar level was observed in heat-treated kiwifruit due to an accelerated ripening process and activation of enzymes [47].

Total phenolic content and flavonoids are the important bioactive compounds and responsible for the total antioxidant activity in harvested fruits and vegetables [23]. Our results demonstrated that use of HWT maintained significantly higher phenolic and flavonoid content in Hillawi (at 65 °C 3 min exposure) and Khadrawi (at 65 °C for 5 min) date cultivars. Saltveit [48] observed that HWT noticeably delayed browning by enhancing phenolic metabolism and reducing the production of phenylalanine ammonia lyase in fresh-cut tissues. On the other hand, Ghasemnezhad et al. [49] reported that HWT induces higher phenolic content in mandarin by improving the enzymes activities. Our results are also in accordance with earlier studies, which documented that HWT maintained maximum phenolic and flavonoid content in peach [50], muskmelon [51], and fresh-cut apple [23]. Previously, it has been reported that HWT at 52 °C for 2 min significantly increased the flavonoid content in kumquat (Schirra et al., 2008) [52]. Likewise, Lafuente et al. [53] reported a higher level of flavonoid content in Fortune mandarin in response to HWT (37 °C). The higher phenolic and flavonoid content in Hillawi and Khadrawi cultivars might be ascribed to upregulation of enzymes activities.

In the current experiment, HWT increased DPPH scavenging activities in date cultivars. However, tannin contents markedly decreased in both date cultivars subjected to HWT. Generally, increased DPPH scavenging activity is directly correlated with higher levels of non-enzymatic antioxidant defense. Similar to our study, it was reported in persimmon [54] and fresh-cut apple [23] that HWT in combination with calcium chloride and calcium ascorbate significantly enhanced the DPPH scavenging activity. The improved antioxidant potential in persimmon and fresh-cut apples subjected to HWT has been attributed to facilitating the entry of calcium content in cytosol [23,54]. Our findings are also in agreement with previous studies that demonstrated that HWT maintained significantly higher total antioxidants in pomegranate [55] and strawberry [56]. Contrary to our results, Naser et al. [54] reported that HWT in combination with calcium lactate reduced the decline in soluble tannin content, which might be due to the membrane-stabilizing effect of calcium.

In general, color, taste, texture, astringency, and overall acceptance are the crucial aspects of fruit and vegetables’ overall sensory quality. Our results indicated that hot water-immersed date cultivars, i.e., Hillawi (at 65 °C for 3 min) and Khadrawi (at 65 °C for 5 min), exhibited better fruit color, and significantly higher sensory quality might be attributed to the regulation of the ripening process and activation of heat shock protein. Our results are in line with those of Wang [57], who reported that hot water-treated kale and collard vegetables showed higher visual quality. Similarly, hot water-dipped cucumber fruit displayed a better appearance and higher sensory quality due to the induction of heat shock protein [58]. Results from our study are also similar to earlier findings reporting higher sensory quality in hot water-treated tomatoes [59], mango [28], and Chinese cabbage [60]. Fruit firmness is one of the most widely used indicators of fruit quality. Lower firmness in hot water-treated Hillawi and Khadrawi date cultivars may be attributed to degradation of pectin substances. Pectin is an essential component of cell walls and solubilization of pectin contents significantly reduces the membrane integrity and results in loss of firmness [61].

## 5. Conclusions

The current study demonstrated that HWT maintained the quality of both cultivars of date palm (Hillawi and Khadrawi) when exposed to 65 °C. HWT effectively shortened the time to reach the tamar stage, expedited ripening, and improved bioactive compounds in Hillawi and Khadrawi date cultivars compared to controls. Moreover, the use of HWT promoted moisture loss, softening, and improved the biochemical properties as well as increased the accumulation of sugar contents causing sweetness compared to untreated date cultivars. Furthermore, the HWT application considerably increased the phenolic content and DPPH scavenging potential, and flavonoid content, while also enhancing the sensory qualities of both date cultivars. Therefore, the application of HWT is a promising technique to stimulate ripening and to maintain and improve fruit quality and appearance of dates.

## Figures and Tables

**Table 1:** Effect of HWT on reducing sugar (a) and total sugar (b) on date palm cvs. Hillawi and Khadrawi.

Reducing Sugars ^a^	Hillawi	Khadrawi
Control	33.14 ± 1.12 g	30.03 ± 3.01 g
HWT-1 min	62.79 ± 3.02 b	40.29 ± 2.85 e
HWT-3 min	69.83 ± 2.05 a	49.12 ± 1.98 de
HWT-5 min	55.96 ± 1.54 c	57.01 ± 2.08 bc
HWT-7 min	47.43 ± 2.63 e	54.95 ± 2.63 cd
Total sugars ^b^		
Control	34.47 ± 1.32 g	31.14 ± 2.14 g
HWT-1 min	65.53 ± 2.65 b	41.53 ± 2.31 f
HWT-3 min	73.02 ± 2.01 a	50.81 ± 2.41 de
HWT-5 min	58.45 ± 1.85 c	59.71 ± 3.05 bc
HWT-7 min	49.26 ± 2.23 e	57.39 ± 2.35 cd

The data collected indicate the means of three replicates, and the standard error indicates the means of replication. The mean values with different letters show significant differences, and same letters indicate no statistically significant difference according to the LSD test (*p* < 0.05).

## Data Availability

The data presented in this study are available on request from the corresponding author.
